# Mesenchymal Stem Cells Exploit Extracellular Matrix as Mechanotransducer

**DOI:** 10.1038/srep02425

**Published:** 2013-08-13

**Authors:** Bojun Li, Cameron Moshfegh, Zhe Lin, Jörg Albuschies, Viola Vogel

**Affiliations:** 1 Department of Health Sciences and Technology, ETH Zurich, Ch-8093 Zurich, Switzerland; 2 These authors contributed equally to this work.

## Abstract

While stem cells can sense and respond to physical properties of their environment, the molecular aspects how physical information is translated into biochemical signals remain unknown. Here we show that human mesenchymal stem cells (hMSCs) harvest and assemble plasma fibronectin into their extracellular matrix (ECM) fibrils within 24 hours. hMSCs pro-actively pull on newly assembled fibronectin ECM fibrils, and the fibers are more stretched on rigid than on soft fibronectin-coated polyacrylamide gels. Culturing hMSCs on single stretched fibronectin fibers upregulates hMSC osteogenesis. Osteogenesis was increased when αvβ3 integrins were blocked on relaxed fibronectin fibers, and decreased when α5β1 integrins were blocked or when epidermal growth factor (EGF) receptor signaling was inhibited on stretched fibronectin fibers. This suggests that hMSCs utilize their own contractile forces to translate environmental cues into differential biochemical signals by stretching fibronectin fibrils. Mechanoregulation of fibronectin fibrils may thus serve as check point to regulate hMSC osteogenesis.

Human mesenchymal stem cells (hMSCs) are adult multipotent stem cells residing within the bone marrow and capable of differentiating into cell types of mesodermal origin including osteogenic, chondrogenic, adipogenic and myogenic lineages^1^, as well as into neurons^2^, vascular endothelial cells^3^ and cardiomyocytes^4^. Due to this diverse differentiation potential, great efforts are underway to exploit hMSCs for regenerative therapies^5^,^6^. One major unsolved challenge is how to guide the specific differentiation of hMSCs in engineered environments and under physiological conditions^6^. Besides biochemical stimuli, various physical properties of synthetic materials have been shown to correlate with hMSC differentiation, including adhesive micro-patterns that dictate cell shape^7^, as well as substrate rigidity^8^ and nanotopography^9^,^10^. Among these physical properties, rigidity gained significant attention after it had been shown that protein-coated polyacrylamide gels with variable rigidities guide the differentiation of hMSCs, independently of soluble factors^8^. In general, cells do not contact the polymer directly, but they are only in physical contact with the adhesive protein layer that is covalently coupled to the surface of the polymer. A recent study now revealed that keratinocytes and hMSCs do not feel the rigidity of a polymer *per se*, but respond to the mechanical feed-back provided by the protein coating which is differently anchored to the underlying polymer network architecture of soft and rigid polyacrylamide gels^11^. The mechanics by which the protein layer is coupled to the polymer thus correlate with stem cell fate. These findings revitalized the discussions about how physical parameters of the combined system (i.e. the materials coupled to the protein coating) are recognized by cells and how they might impact their functions^12^,^13^,^14^. Encapsulation of murine MSCs in 3D hydrogels with different rigidities furthermore suggested that MSCs might respond to their increased ability to cluster integrin ligands in flexible environments^12^. Furthermore, it has never been investigated whether the early ECM that cells assemble in response to the effective mechanical properties of substrate plays an active role in translating physical environmental cues into biochemical signals that ultimately regulate stem cell differentiation, and whether early exposure to ECM fibrils might alter this behavior.

Fibrillar fibronectin (Fn) is the first ECM that is actively assembled by many cell types upon injury, either by harvesting plasma Fn or by producing their own^15^. It plays a crucial role in wound healing, as well as during the healing of tissues in contact with implants and in early embryonic development^16^. Cells can remodel ECM or their surrounding protein environment through the generation of traction forces which leads to the stretching of Fn fibrils^17^. Stretching of Fn fibrils may activate cryptic binding sites^18^,^19^,^20^ or destroy binding motives that are only displayed on unstretched fibers (Fig. 1), including the multivalent binding motifs on fibronectin for bacterial adhesins^21^. Our study builds upon the observation that the conformation of surface-bound Fn can impact osteogenic differentiation^22^,^23^,^24^, and that the tension of early Fn ECM fibrils assembled by fibroblasts is significantly higher when the cells can pull on a protein layer that is covalently linked to rigid compared to soft polyacrylamide surfaces as probed by fluorescence resonance energy transfer (FRET)^25^.

Here we asked whether Fn fibrils in early ECM play a mechano-regulatory role by translating microenvironmental inputs into biochemical signals that might regulate hMSC differentiation. Since stretching of Fn fibers changes their rigidity^19^, as well as their biochemical display of binding sites^19^,^20^,^21^,^26^, a single Fn fiber stretch assay was prepared to distinguish the differential effects of fiber rigidity versus Fn's biochemical display on the differentiation of hMSCs^27^.

## Results

### hMSCs stretch their own fn fibrils in early ECM more on rigid than on soft polyacrylamide gels

Alterations of the rigidity of protein-coated polyacrylamide gels have been shown to correlate with hMSC differentiation^8^, but the mechanism remains unknown. To investigate how hMSCs translate physical properties of their environment into a biochemical signal that regulates differentiation, hMSCs were seeded at a low seeding density (3 × 10^3^/cm^2^) on a layer of Fn that was covalently linked to polyacrylamide gels of different rigidities. The hMSCs were cultured for 1 day in mixed induction medium (50/50 vol% adipogenic/osteogenic induction medium), which contained trace amounts of FRET labeled Fn. hMSCs were able to assemble Fn ECM within the first 24 hours on polyacrylamide gels (Fig. 2a–c (on polyacrylamide)) and on glass (Fig. S1).

**Figure 1:**
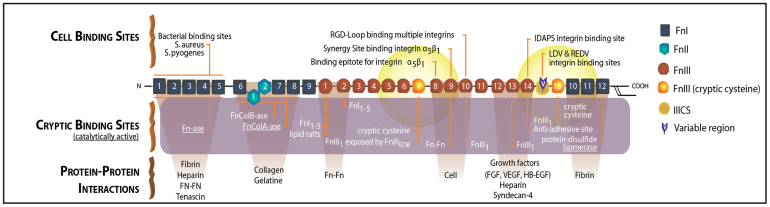
Schematic structure of monomeric plasma fibronectin with its binding sites. Fn contains a large number of cell binding and protein-protein interaction sites, including the famous cell binding site RGD^28^ on FnIII_10_ and the synergy site PHSRN on FnIII_9_^29^,^30^_._ Many cryptic binding sites were also detected in Fn, including various Fn self-assembly sites whose exposure is needed to induce Fn fibrillogenesis (as reviewed in^26^,^31^). It is proposed that the cryptic binding sites might be exposed when cells stretch Fn, while some of the other sites that are exposed under equilibrium might get destroyed through loss of secondary structure^21^,^26^. Two cryptic, non-disulfide bonded cysteines in FnIII_7_ and FnIII_15_ (shown with orange color) were used in our studies for FRET-labeling using Alexa 546 as acceptor, and about 3.5 amines per monomeric Fn were randomly labeled with Alexa 488 as donor. The Förster radius of this fluorophore pair is ~6 nm (from Invitrogen). Hence the energy transfer is limited to within 12 nm of FnIII_7_ and FnIII_15_ (yellow fading spheres). Adapted from^26^.

**Figure 2:**
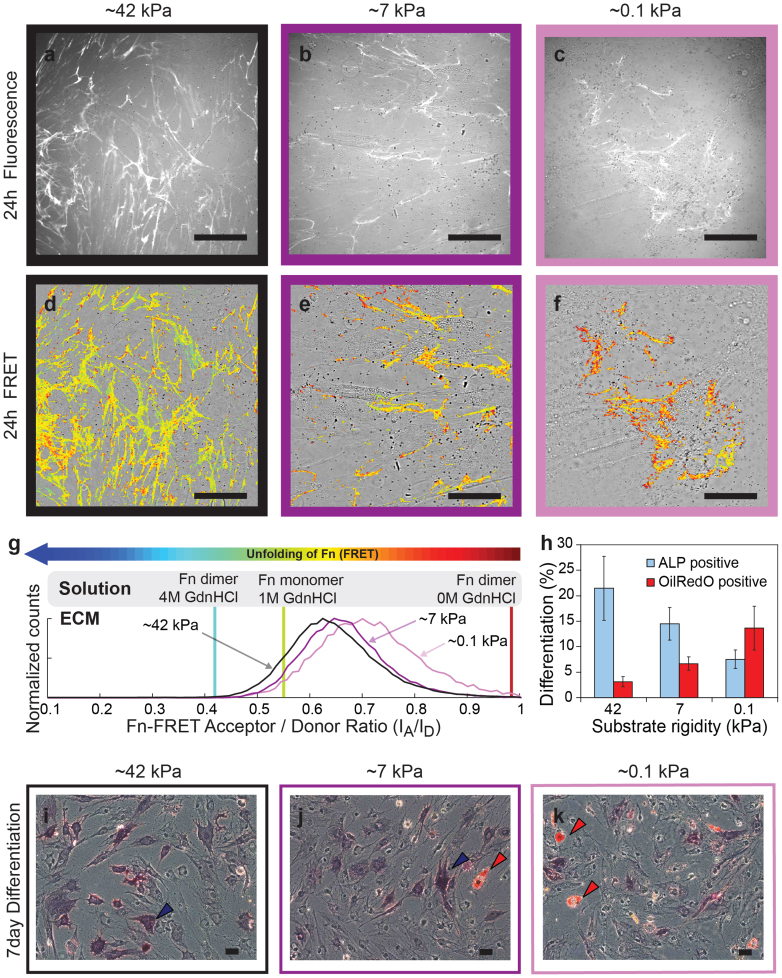
The bulk rigidity of polyacrylamide gels correlates with the osteogenic differentiation of hMSCs, as well as with the Fn strain of early ECM. (a–c) Merged images of hMSC-assembled Fn fibrils in early ECM (fluorescence image) with brightfield images of hMSCs cultured for 24 hours on rigid (a, ~42 kPa), medium (b, ~7 kPa) or soft (c, ~0.1 kPa) Fn-functionalized polyacrylamide gels at a seeding density of 3 × 10^3^ cells/cm^2^ in mixed induction medium supplemented with Fn-FRET. (d–f) Ratiometric Fn-FRET I_A_/I_D_ images. Merged images of hMSC-assembled 24 hour Fn ECM (FRET false colors) with brightfield images of hMSCs cultured on rigid (d), medium (e) or soft (f) Fn-functionalized polyacrylamide substrates at a seeding density of 3 × 10^3^ cells/cm^2^ for 24 hours in mixed induction medium supplemented with Fn-FRET. The FRET false color scheme represents the relative stretching of Fn fibrils with a color range of red to blue indicating folded to unfolded states of Fn, respectively. (g) Histograms of Fn-FRET I_A_/I_D_ ratios of hMSC-assembled Fn ECM on rigid (black curve), medium (purple curve) or soft (pink curve) Fn-functionalized polyacrylamide substrates after 24 hours. (h) Percentage of OilRedO and ALP positive hMSCs. Results are shown as the mean ± s.d. (n = 3). (i–k) Brightfield images of hMSCs cultured on rigid (i), medium (j) or soft (k) Fn-functionalized polyacrylamide gels at a seeding density of 3 × 10^3^ cells/cm^2^ for 7 days in mixed induction medium supplemented with trace amounts of Fn-FRET, with histochemical staining for ALP (blue, blue arrows) and OilRedO (red, red arrows). Scale bars: 50 μm.

To be able to probe the Fn fiber strain, Fn was labeled for FRET with donors and acceptors (Fig. 1). Briefly, the native cysteines on FnIII_7_ and FnIII_15_ of human plasma Fn were site-specifically labeled with Alexa Fluor 546 acceptors (A), whereas amines were randomly labeled with Alexa Fluor 488 donors (D)^17^,^32^. The relative emission intensities of the FRET pair were quantified by calculating the ratio of measured acceptor to donor intensities (I_A_/I_D_). Local stretching of Fn fibrils by cellular traction forces leads to an increase of the average distance between acceptor and donor fluorophores and thus to a reduction in FRET (I_A_/I_D_ ratio)^32^. Fn-FRET I_A_/I_D_ ratios were color-coded within the range of 0.05 to 1.0 to yield FRET ratio-images (Fig. 2d–f). The FRET false color scheme represents the relative stretching of Fn fibrils with a color range of red to blue indicating folded to unfolded states of Fn, respectively. As shown in Fig. 2g, the FRET ratio was lowest on rigid substrates, and increased with decreasing rigidity. This change of Fn strain was confirmed by analysis of the average FRET I_A_/I_D_ ratios of 10 Fn matrices (Fig. S2).

After culturing the hMSCs in mixed induction medium supplemented with traces of Fn-FRET for 7 days, hMSC osteogenic and adipogenic differentiation were checked by histochemical staining for ALP and OilRedO, respectively. As previously described^7^ and confirmed here, hMSCs can differentiate into adipogenic and osteogenic cell lineages in mixed induction medium after 7 days culture (Fig. S3). Within the reported rigidity sensing regime, from 0.1 kPa to 42 kPa^8^, we found that osteogenic differentiation of hMSCs as probed by ALP staining was upregulated with increasing substrate rigidity. The hMSC osteogenic differentiation correlated well with the rigidity of Fn-coated polyacrylamide gels (Fig. 2h, i–k) as reported before^8^. We describe for the first time that the changes in the rigidity of fibronectin-coated polyacrylamide gels correlate with significant shifts in the Fn-FRET ratios within early ECM that hMSCs produce. The same correlations between Fn fibril tensions and polyacrylamide rigidities were also observed on collagen I-coated polyacrylamide gels (Fig. S4). Consequently, the rigidity of polyacrylamide gels coated with a layer of adhesion proteins not only correlated with stem cell differentiation, but also with the mechanical strain of early Fn fibrils assembled by the still undifferentiated hMSCs within the first few hours. This is in agreement with previous reports showing that fibroblasts pull more strongly on their Fn fibrils of early ECM when cultured on rigid versus soft polyacrylamide gels^25^.

In contrast, the same correlations between Fn fibril strains and altered polymer rigidity cannot be seen for hMSCs cultured on PDMS surfaces where a layer of either Fn or collagen I was covalently bound to the substrate. The mechanical strain of early ECM Fn fibrils assembled on Fn (Fig. S5) or collagen I (Fig. S6) covalently coated PDMS substrates with varying rigidities remained constant. In contrast to polyacrylamide, PDMS does not have a porous nanoscale architecture which leads to a much more homogenous anchorage of the protein layer^11^. Remarkably and in agreement with our previous report, the altered rigidity of PDMS did not influence hMSC differentiation^11^. Taken together, it is not substrate rigidity of the polymer *per se* that correlates with the upregulation of osteogenic differentiation. Instead and most importantly, variations of substrate rigidity affected the differentiation of hMSCs only in those cases where the mechanical strain of early Fn fibrils was altered too.

### Also stretched single Fn fibers used as substrates upregulate osteogenic differentiation of hMSCs

**Figure 3:**
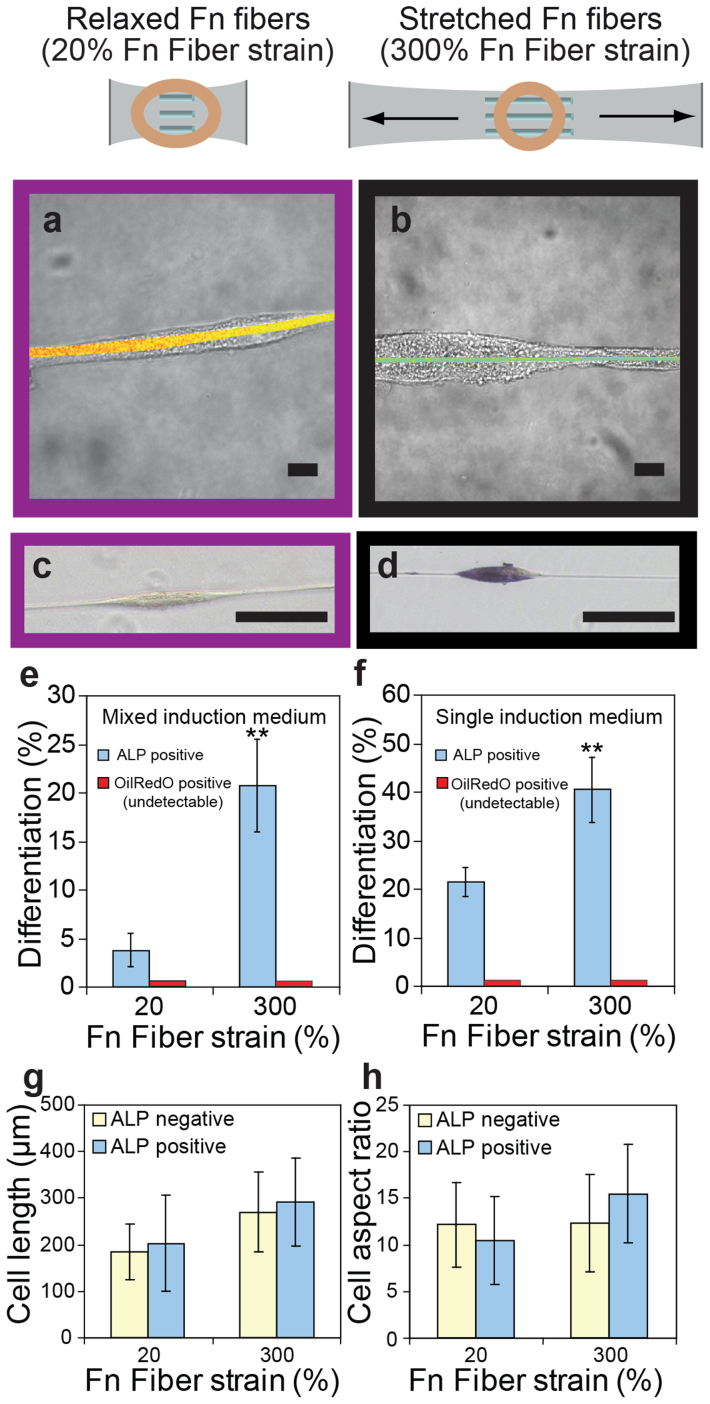
Osteogenic differentiation of hMSCs is upregulated on stretched single Fn fibers. (a and b) Brightfield images of hMSCs merged with Fn-FRET images of relaxed (a) or stretched (b) single Fn fibers after cell attachment and culture for 2 days (Scale bars: 10 μm). (c and d) Brightfield images of hMSCs cultured on crosslinked, relaxed ((c), 20% fiber strain) or stretched ((d), 300% fiber strain) single Fn fibers in mixed induction medium for 7 days, with histochemical staining for ALP (blue). Scale bars: 100 μm. (e and f) Percentage of OilRedO and ALP positive hMSCs when cultured on single Fn fibers in mixed (e) or single (f) induction medium for 7 days. No OilRedO positive hMSCs could be detected on stretched or relaxed Fn fibers. Data shown represent mean ± s.d. (n = 5). Two asterisks: p < 0.01 versus 20% Fn fiber strain (p = 0.0003 for (e), p = 0.0006 for (f)). (g and h) Analysis of cell length (g) and aspect ratio (h) of ALP positive (blue) and ALP negative (yellow) hMSCs on relaxed and stretched single Fn fibers. Data shown represent mean ± s.d. (n = 8).

To test whether hMSC differentiation can be regulated by stretching Fn fibers, hMSCs were cultured on single Fn fibers that were physically coupled to stretchable silicone sheets^27^. The stretching of Fn fibers increases their rigidity^19^ and might simultaneously switch the exposure of molecular binding sites^21^,^26^. However, the rigidity of these deposited Fn fibers was more than 0.8 MPa^33^, and the stiffness of our silicone sheets exceeded 1 MPa (data not shown) which is much higher than the range of cell-sensed rigidities that affect hMSC differentiation^8^. Therefore, physiologically relevant rigidity alterations could be excluded in this system. By stretching the silicone sheets, Fn fibers of relaxed (20% fiber strain^19^) (Fig. 3a) and stretched (300% fiber strain^19^) (Fig. 3b) conformations were prepared, followed by Fn crosslinking with 4% formaldehyde to further reduce potential rigidity differences^33^. Undifferentiated hMSCs adopted a highly elongated spindle shape (Fig. 3a and 3b) and were not able to change the Fn conformation of deposited fibers by tensile forces (Fig. 3a, 3b and Fig. S7).

The ratio of alkaline phosphatase (ALP) positive cells was much higher on stretched fibers than on relaxed fibers. These results are independent of whether the hMSCs were cultured on single Fn fibers either in mixed (50/50 vol% adipogenic/osteogenic induction medium) or pure osteogenic induction medium for 7 days (Fig. 3e and 3f). The experiments were repeated several times. In mixed induction medium, a total of 196 hMSCs on the stretched crosslinked fibers and 120 hMSCs on relaxed crosslinked fibers were analyzed. On stretched crosslinked fibers about 21% of the cells showed osteogenic differentiation, while on relaxed crosslinked fibers only about 4% stained ALP positive. In pure osteogenic induction medium, a total of 210 hMSCs on the stretched crosslinked fibers and 187 hMSCs on relaxed crosslinked fibers were analyzed. About 41% of the cells showed osteogenic differentiation on stretched crosslinked fibers, and about 22% showed osteogenic differentiation on relaxed crosslinked fibers. The same trend was also observed on fibers that had not been crosslinked, or on Fn fibers with 20% (relaxed) and 300% (stretched) strain on the same silicone sheet (Fn fibers were deposited perpendicular to each other onto the same silicone sheet; stretching of the sheet results in stretching and relaxation of fibers at the same time). This confirms that stretch-induced changes in the rigidity of the silicone sheet did not drive the increased hMSC osteogenic differentiation on stretched Fn fibers. Also small changes of cell shape on single Fn fibers did not positively correlate with the differentiation path (Fig. 3g and 3h). These results show that stretched Fn fibers enhance hMSC osteogenesis. Importantly, these findings are not dependent on changes of cell shape (Fig. 3g and 3h) or changes in Fn fiber rigidity. Together with the previous observation that cells pull more strongly on Fn fibers of early ECM fibrils on protein-coated rigid than soft polyacrylamide gels (Fig. 3), this suggests that the mechanical strain of early Fn ECM might be involved in regulating hMSC differentiation.

### Adipogenesis on single Fn fibers was not observed, even in adipogenic culture medium

When hMSCs were cultured on single Fn fibers (Fig. 3c and 3d), no OilRedO positive hMSCs were observed after 7 days, neither in mixed (Fig. 3e) nor in pure adipogenic induction medium (Fig. 3f). Previously it has been shown that cell shape regulated adipogenesis of hMSCs and that a spread cell shape inhibited adipogenesis^7^. hMSCs were forced to assume an elongated cell shape on Fn fibers. hMSCs either did not differentiate into adipocytes while in contact with Fn fibers, or they detached from the fibers.

### Blocking αvβ3 integrin binding upregulates osteogenesis on relaxed fibers, while blocking α5β1 slightly downregulates osteogenesis on stretched Fn fibers

**Figure 4:**
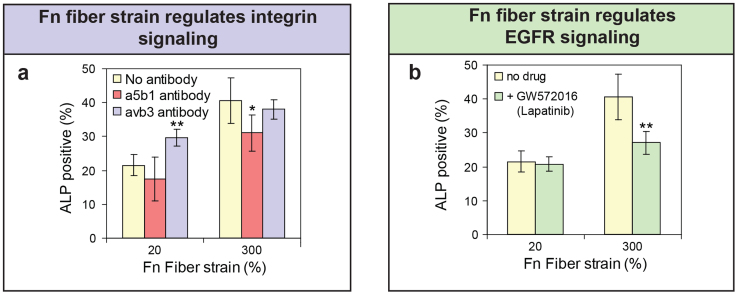
Fn fiber strain regulates differential integrin and EGFR signaling. (a) hMSCs cultured on single Fn fibers in osteogenic induction medium for 7 days with or without constant exposure to function-blocking antibodies. Percentage of ALP positive hMSCs is shown in the presence of function-blocking antibodies against integrin α5β1 (red), integrin αvβ3 (blue) or without antibodies (yellow). (b) Percentage of ALP positive hMSCs when cultured for 7 days on single Fn fibers in the presence (green) or absence (yellow) of the EGFR inhibitor GW572016 in osteogenic induction medium is shown. Data shown in a and b represent mean ± s.d. (n = 5). Asterisk p < 0.05 versus no antibody treatment (P = 0.0385 for α5β1 in (a)). Two asterisks: p < 0.01 versus no antibody treatment (P = 0.0038 for αvβ3 in (a)) or no drug treatment (p = 0.0092 for (b)).

Previous studies on Fn adsorbed to surfaces presenting different chemistries^34^ suggested that differential activation of the two most prominent Fn-binding integrins, α5β1 and αvβ3, may be involved in the regulation of hMSC osteogenesis by Fn conformations. To test for the differential roles of α5β1 versus αvβ3 integrins in recognizing whether Fn fibers are stretched or relaxed, hMSCs were cultured on single Fn fibers in pure osteogenic induction medium (Fig. 4). In the presence of both function-blocking antibodies against α5β1 (clone JBS5) and αvβ3 (clone 23C6), hMSCs were not able to attach to single Fn fibers. This confirmed that the antibodies were active. The continued presence of the αvβ3 antibody alone allowed cell attachment and increased the ratio of ALP positive hMSCs (from 22% to 30%, p < 0.01) on relaxed Fn fibers after 7 days, but did not do so on the stretched Fn fibers (Fig. 4a). Blocking α5β1 binding slightly decreased the ratio of ALP positive hMSCs (from 41% to 31%, p < 0.05) on stretched Fn fibers, but had no effect on hMSCs on relaxed Fn fibers (Fig. 4a). This shows that both integrins are involved in mediating cell attachment and differentially regulate the differentiation of hMSCs on Fn fibers. Importantly, the two integrins show different dependencies to Fn stretching. This suggests that the conformational change of Fn caused by the stretching fibers might regulate integrin binding which could finally impact hMSC osteogenesis on fibers. However, the increase in osteogenesis on stretched Fn fibers could not be fully reverted, neither by blocking of α5β1 nor αvβ3. This suggests that additional mechanisms might exist that regulate the mechanosensitive osteogenesis of hMSCs in contact with mechanically strained Fn fibers. One possible mechanism may be the change of ligand density caused by the stretching of fibers. There is a possibility that stretching of Fn fibers decreases the ligand density on the surface of manually pulled fibers. It was reported that ligand density could impact cell activities^35^,^36^. However, it has not been reported that a decrease in the density of some binding sites of Fn could promote osteogenesis. Also, a change in ligand density alone is unlikely to cause different integrin signaling. Therefore, on manually pulled Fn fibers, different integrin signaling caused by stretching of fibers may combine with other mechanisms to regulate hMSC osteogenesis.

### Stretch-induced upregulation of osteogenesis on single Fn fibers is decreased upon inhibition of the EGF receptor (ErbB)

Integrins have been reported to cooperate synergistically with growth factor receptors to regulate cell activities^37^,^38^. For example, integrin α5β1 mediates Fn-dependent epithelial cell proliferation through epidermal growth factor (EGF) receptor activation^39^, and potent synergistic signaling between α5β1 integrin and the growth factor receptors when the integrin and growth factor binding sites are presented at the right distance from each other^37^. The ErbB family of transmembrane receptors belongs to the epidermal growth factor (EGF) receptor family of receptor tyrosine kinases. Since it was recently shown that ErbB3 and ErbB4 (also called HER3 and HER4) are involved in the regulation of the rigidity response of fibroblasts that were spreading on PDMS substrates coated with adsorbed (not crosslinked) Fn^40^, we tested whether ErbBs are also involved in the mechanosensing of Fn fiber strain. hMSCs were cultured on single Fn fibers for 7 days in pure osteogenic induction medium containing the ErbB inhibitor GW572016 (Lapatinib)^41^. GW572016 treatment only decreased the ratio of ALP positive hMSCs on stretched Fn fibers (from 41% to 27%, p < 0.01), but had no effect on the differentiation on relaxed Fn fibers (Fig. 4b). These results imply that ErbBs by themselves or downstream signaling are indeed involved in distinguishing between stretched and relaxed Fn fibers.

## Discussion

Based on many previous studies performed on flat polymeric surfaces or in hydrogels^7^,^8^,^12^,^42^, it was demonstrated that physical properties of biomaterials can correlate with hMSC osteogenesis, but the underlying mechanism remains unknown. To shed light into the question of how cells sense physical properties of synthetic materials, we investigated whether the early ECM that cells assemble on materials plays an essential role in translating physical properties into biochemical signals that can regulate cell function. We showed here that hMSCs assembled early ECM by harvesting plasma Fn from the medium within the first 24 hours, and stretched their own Fn ECM fibrils (Fig. 2). Importantly, hMSCs could regulate the strain of the early Fn ECM by stretching the Fn ECM fibrils more on protein-coated rigid than on soft polyacrylamide gels (Fig. 2). Manually stretched Fn fibers upregulated hMSC osteogenesis independently of cell shape and fiber rigidity (Fig. 3). In contrast to polyacrylamide hydrogels whose nanoscale surface architecture is altered as function of its bulk rigidity^11^, we would like to propose that smooth PDMS substrates with varying rigidities did not impact hMSC differentiation because the rigidity variations here did not affect the Fn strains in early ECM (Fig. S5 and S6). This suggests that hMSCs exploit the strain of Fn ECM fibrils, which they self-adjust by their own traction forces, as a mechano-regulated check point regulating hMSC osteogenic differentiation in response to the effective mechanical properties of protein-coated substrates.

We further investigated possible molecular mechanisms by which the stretching of Fn fibers regulates hMSC differentiation. Differential integrin α5β1 and αvβ3 binding is shown here to be mechano-regulated by adjustments of Fn fiber strain (Fig. 4a). Previous studies suggest differential integrin signaling as the major mechanism by which altered Fn conformations might regulate outside-in signaling^24^,^43^, and potentially osteogenesis^22^. As expected, integrin signaling is differentially affected by stretching Fn fibrils. The continued presence of the αvβ3 function blocking antibody increased the ratio of ALP positive hMSCs on relaxed Fn fibers after 7 days, but did not do so on the stretched Fn fibers (Fig. 4a). In contrast, inhibition of Fn-α5β1 binding slightly reduced the ratio of ALP positive hMSCs on stretched but not on relaxed single Fn fibers (Fig. 4a). This suggests that relaxed Fn fibers preferentially signaled through integrin αvβ3 over α5β1, resulting in decreased hMSC osteogenesis on relaxed fibers. This finding was counterintuitive since the stretch-induced conformational changes of Fn which regulate integrin binding may involve the elongation of the relative distance of the synergy site on FnIII_9_ with respect to the RGD-loop on FnIII_10_ as previously reported^34^,^43^. We surprisingly found that blocking either of the two integrins still showed a relatively weak effect compared to the stretch-induced increase of hMSC osteogenesis on Fn fibers (Fig. 4a). This gave a first hint towards the existence of additional outside-in signaling cascades that are differentially regulated by Fn fiber strain.

In addition to integrin signaling, a pronounced effect was seen here upon pharmaceutical inhibition of the EGF growth factor receptor ErbB by GW572016 treatment: a decreased ratio of ALP positive hMSCs was seen on stretched Fn fibers, but had no regulatory effect on the osteogenesis of hMSCs on relaxed Fn fibers (Fig. 4b). These results thus imply that ErbBs, or ErbB-affected downstream signaling events, are also involved in distinguishing between stretched and relaxed single Fn fibers. GW572016 not only inhibits ErbB1 but also other ErbB members, including ErbB2^41^, ErbB3^44^ and ErbB4^45^. Among the ErbB family, ErbB3 and ErbB4 are reported to be involved in the regulation of the response of fibroblasts to PDMS substrate rigidity^40^, but the mechanism is unknown. For hMSC osteogenic differentiation, it has been shown that a sustained activation of ErbB1 by surface-tethered EGF increases hMSC osteogenesis via the MAPK/ERK pathway^46^. ErbB2 has no ligand^47^, but instead heterodimerizes with other ErbBs such as ErbB1^48^, and sustained activation of ErbB1 and ErbB2 enhances hMSC osteogenesis^49^. It is unknown whether ErbB receptors are directly associated with Fn fiber strain or whether ErbB-affected downstream signaling is involved. Further studies are clearly needed to gain more detailed insights into the role of EGF and its receptors (ErbBs) in translating the mechanical strain of Fn fibers into an osteogenic response. Finally, FnIII_12-14_ fragments were found previously to bind promiscuously to many growth factor families, including members of the PDGF, FGF, TGF-β families and heparin binding-EGF (HB-EGF)^50^. While shown here for EGF receptor-mediated signaling, also other growth factor receptors might thus contribute to sensing the mechanical strain of extracellular matrix fibers.

In summary, we now propose a mechanism by which hMSCs can sense the effective physical properties of synthetic materials coated with a protein layer:

hMSCs can harvest plasma Fn from the medium and assemble it into an early provisional ECM already within the first 24 hours (Fig. 2). They stretch the Fn fibrils within early ECM considerably more on protein-coated rigid than on soft polyacrylamide gels (Fig. 2)^8^. The stretched Fn fibrils promote hMSC osteogenesis through differential biochemical signaling which may involve αvβ3 and α5β1 integrins and ErbB signaling pathways (Fig. 4). Thus before the hMSCs start to upregulate the expression of their own Fn^51^, hMSCs can start to exploit Fn harvested from the medium or serum as a mechanotransducer to self-adjust the functional display of ECM fibrils.

Taken together, the data suggest that Fn serves as pivotal mechano-chemical signal converter, and that its mechanical strain might serve as a check point by which hMSCs can translate physical aspects of their environment into biochemical signals that direct hMSC differentiation. The translation of different physical properties may involve a larger set of transmembrane receptors, including integrins and ErbBs. Learning how hMSCs exploit the tension of ECM fibrils to self-regulate their differentiation path is crucial to initiate new ideas how to exploit MSCs for therapeutic purposes, or for the design of advanced biomimetic scaffolds for tissue engineering applications.

## Methods

### Cell culture

hMSCs (Lonza) were cultured as specified, either in growth medium (DMEM, 10% FBS, 0.3 mg/ml glutamine, 100 units/ml penicillin and 100 μg/ml streptomycin), osteogenic, adipogenic or mixed (50/50 vol% adipogenic/osteogenic) induction medium (Lonza). Only early passage hMSCs (up to passage 5) were used. To inhibit specific integrin-Fn interactions, hMSCs (50 × 10^3^ cells/ml) were allowed to attach for 1 hour on single Fn fibers in growth medium containing function-blocking antibodies against either integrin α5β1 (10 μg/ml, clone JBS5, Abcam) or αvβ3 (10 μg/ml, clone 23C6, Abcam), followed by culture in osteogenic induction medium containing the respective integrin function-blocking antibody for 7 days. To block the activity of ErbB receptors, hMSCs (50 × 10^3^ cells/ml) were allowed to attach for 1 hour on single Fn fibers in growth medium supplemented with GW572016 (1 μM, Lapatinib, Axon Medchem), and were subsequently cultured for 7 days in osteogenic induction medium supplemented with GW572016.

### Fn labeling with FRET donors/acceptors and FRET analysis

The following methods were deployed to study the mechanoregulatory role of Fn in directing hMSC differentiation. Fn's conformation was monitored by adding small amounts of FRET-labeled Fn (Fn-FRET) as mechanical strain probe to the cell medium^32^,^52^. The native cysteines on FnIII_7_ and FnIII_15_ were site-specifically labeled with Alexa Fluor 546 acceptor fluorophores (A), whereas amines were randomly labeled with Alexa Fluor 488 donor fluorophores (D). Local stretching of Fn fibrils by cellular traction forces leads to an increase of the average distance between acceptor and donor fluorophores and thus to a reduction in FRET (I_A_/I_D_).

Fn, purified from human plasma (Swiss Red Cross)^53^, was doubly labeled with Alexa Fluor 488 succinimidyl ester and Alexa Fluor 546 maleimide (Molecular Probes) as FRET donors and acceptors respectively, as previously described^32^. The labeling ratio of Fn-FRET was determined by measuring the absorbance of Fn-FRET at 280, 498 and 556 nm and using extinction coefficients of 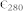 = 8′789 M^−1^cm^−1^, 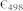 = 78′000 M^−1^cm^−1^ and 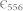 = 0 M^−1^cm^−1^ for Alexa Fluor 488, 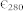 = 12′500 M^−1^cm^−1^, 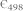 = 13′000 M^−1^cm^−1^ and 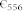 = 105′000 M^−1^cm^−1^ for Alexa Fluor 546 (http://www.invitrogen.com/) and 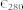 = 563′200 M^−1^cm^−1^ for Fn^54^,^55^. The two-step labeling resulted in an average of 7.1 donors and 3.8 acceptors on each Fn dimer. Fn-FRET was stored as 10 μl aliquots in PBS at −20°C and used within 5 days upon thawing. The same batch of Fn-FRET was used for all FRET data shown in this paper.

### FRET analysis

All images were acquired using an Olympus (http://www.olympus-global.com/) FV-1000 scanning laser Confocal microscope with a 1.35NA 60× oil immersion objective. Alexa Fluor 488 donors of the Fn-FRET were excited with a 488 nm laser. Emitted light was split using a 50/50 beam splitter and detected in two separate photomultiplier tubes (PMTs). Emission detection windows were set at 514–526 nm (donor channel) and 566–578 nm (acceptor channel) to capture peak emissions. Images were acquired at a resolution of 512 × 512 pixels for a 212 × 212 μm field of view with a pinhole diameter of 200 μm. The images were analyzed using Matlab (http://www.mathworks.com/) according to a previous script^32^. First, images were averaged with 2 × 2 pixel sliding blocks, and the dark current background was subtracted from donor and acceptor images (previously acquired for each experiment). Donor images were corrected for light attenuation from the 50/50 beam splitter with a multiplication factor of 1.09. A threshold mask of 100 relative intensity units was applied to both images and the acceptor image was divided pixel by pixel by the donor image for all pixels above threshold intensity values to yield Fn-FRET I_A_/I_D_ ratios. Decreasing Fn-FRET I_A_/I_D_ ratios indicated more extended Fn conformations. Histograms were computed from all data pixels within each field of view and Fn-FRET I_A_/I_D_ ratios were color-coded within the range of 0.05 to 1.0 to produce FRET images. For each sample, histograms were also collected from 10 randomly chosen images showing in all cases that the histograms given in Fig. 2 for single image is representative. Brightfield images were background subtracted using a polynomial fit (degree of 32) with the ImageJ software (http://rsbweb.nih.gov/ij/).

### Chemical denaturation curve of Fn-FRET in solution

Fn-FRET I_A_/I_D_ ratios were calibrated to different Fn conformations in solution for monomeric and dimeric Fn-FRET in different concentrations of denaturant^32^. Solutions of dimeric Fn-FRET in 0 M, 1 M, 4 M GdnHCl and monomeric Fn-FRET in 1 M GdnHCl were added into 2 mm wide chambers on Bovine serum albumin (BSA, Sigma-Aldrich) coated glass coverslips separated by 0.25-mm-thick silicone sheets (Specialty Manufacturing, Saginaw, MI). Fn-FRET solutions (about 2 μl) were imaged using the same scanning laser Confocal microscope (Olympus FV-1000, http://www.olympus-global.com/).

### Preparation of manually deposited single Fn fibers deposited on silicone sheets

Based on a previous procedure^27^, 0.25-mm-thick silicone sheets (Specialty Manufacturing, Saginaw, MI) were cut into 4 × 1.7 cm rectangles, plasma cleaned and fixed on an autoclaved strain device. A sharp-edged plastic pipette tip was used to pull Fn fibers from a 0.4 mg/ml Fn solution in PBS (5% Fn-FRET and 95% unlabeled Fn) on the silicone sheet. Pulled Fn fibers were washed and kept wet with PBS. After Fn fiber deposition, relaxed Fn fibers were prepared by relaxing the silicone sheet to 0.5 times the initial length (corresponding to 20% fiber strain^19^), while stretched Fn fibers were prepared by stretching the silicone sheet to 1.7 times the initial length (corresponding to 300% fiber strain^19^). After stretching or relaxation, Fn fibers were crosslinked with 4% formaldehyde for 1 hour. The silicone sheets were backfilled with 0.1 mg/ml PLL(20)-g(3.5)-PEG(2) for 1 hour and rinsed with PBS.

### Cell staining

ALP was stained using the Sigma kit #85 according to the manufacturer's protocol. For the staining of lipids, cells were fixed with 10% formaldehyde and rinsed with 60% isopropanol. Cells were then stained with 30 mg/ml OilredO (Sigma) in 60% isopropanol. Cells were stained with 3 μg/ml DAPI (Invitrogen) to visualize cell nuclei. Cells were photographed and counted using an Axiovert 200 M inverted microscope (Carl Zeiss).

### Preparation of 2D polyacrylamide substrates

In accordance with a previously described protocol^25^, 35 mm glass-bottom dishes were plasma cleaned, silanized using aminopropyltriethoxysilane and treated with glutaraldehyde. The surfaces were coated with 10 μl droplets of 10% polyacrylamide/0.26% bisacrylamide for the ~42 kPa rigid substrate (41.8 kPa +/−10.7 kPa), 10% polyacrylamide/0.05% bisacrylamide for the ~7 kPa medium-stiff substrate (7.3 kPa +/−0.6 kPa) or 3% polyacrylamide/0.05% bisacrylamide for the ~0.1 kPa soft substrate (0.13 kPa +/−0.005 kPa) and covered with 12 mm diameter coverslips. Coverslips were removed and the polyacrylamide surfaces covalently functionalized with Fn or collagen I using sulfosuccinimidyl-6 (4′-azido-2′-nitrophenylamino) hexanoate (sulfo-SANPAH, Pierce) to allow cell attachment. Briefly polyacrylamide gels were placed in a 24-well plate and 500 μl of a 0.2 mg/ml solution of sulfo-SANPAH in milli-Q H_2_O were added to each well. The PDMS surface was irradiated for 5 minutes using the 365 nm UV LED array. The solution was removed and the procedure was repeated once. After washing with 50 mM HEPES in PBS (twice), the substrates were coated with 50 μg/ml collagen I or 20 μg/ml Fn (purified by ourselves) in PBS. The Young's moduli of the polyacrylamide gels were determined by atomic force microscopy (AFM) using a silicon nitride tip with an attached polystyrene bead (Novascan, 4.5 μm bead diameter, 10 pN/nm spring constant) and a modified Hertz model as previously described^56^. The AFM-derived Young's moduli were in good agreement with recent literature values of comparable polyacrylamide gel compositions^57^.

### Preparation of 2D PDMS substrates

In accordance with a previously described protocol^11^, 13 mm glass coverslips (thickness no 1, borosilicate glass) were thoroughly cleaned by ultrasonication in milli-Q H_2_O and ethanol. The two parts of the PDMS kit (Sylgard 184, VWR) were mixed in different ratios ranging from 100:1 to 10:1 base:crosslinker and spread on the glass coverslips. The elastomers were cured at 70°C overnight. For cell seeding, Fn or collagen I was covalently attached to the PDMS surface using the same sulfo-SANPAH linker as for the Polyacrylamide hydrogels.

### Statistical analysis

Statistical differences between two groups of data were analyzed with Student's t-test. Data are presented as means ± s.d.

## Author Contributions

B.L., C.M. and V.V. designed the research and B.L., C.M. and Z.L. performed it. B.L., C.M., Z.L. and V.V. analyzed the data. J.A. performed AFM indentation measurements and analysis. B.L., C.M. and V.V. wrote the paper.

## Supplementary Material

Supplementary Information Mesenchymal Stem Cells Exploit Extracellular Matrix as Mechanotransducer
