# Supplementary material for: Mesenchymal Stem Cells Exploit Extracellular Matrix as Mechanotransducer
Source: Sci Rep. 2013 Aug 13;3:2425. doi: 10.1038/srep02425 (PMC3741624; doi:10.1038/srep02425)
Supplement: Supplementary Information — Mesenchymal Stem Cells Exploit Extracellular Matrix as Mechanotransducer [file srep02425-s1.pdf]

Supplemental Information

Mesenchymal Stem Cells Exploit Extracellular Matrix as Mechanotransducer

Bojun Li, Cameron Moshfegh, Zhe Lin, Jörg Albuschies and Viola Vogel

Inventory of Supplemental Information

Supplemental Figures

Supplemental References

Supplemental Figures

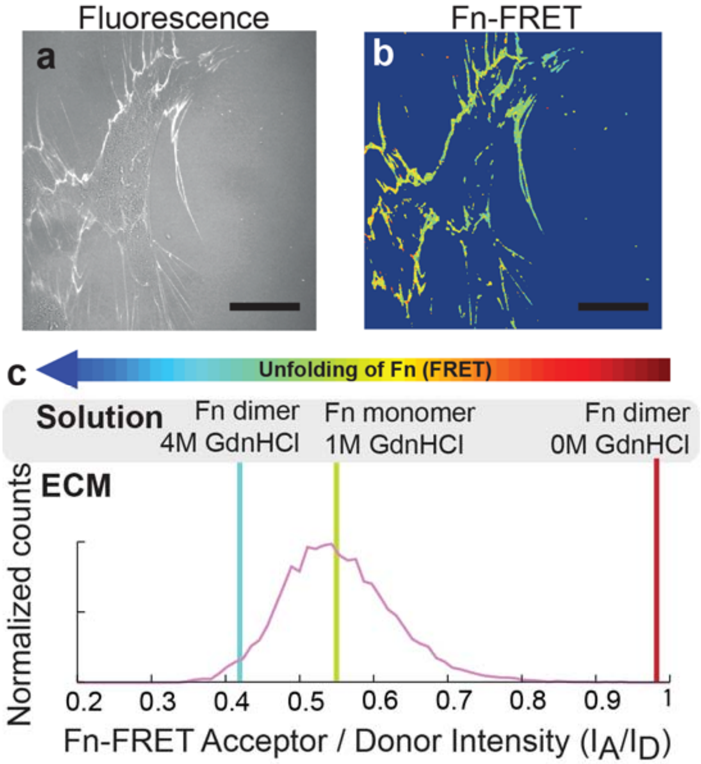

Figure S1: Undifferentiated hMSCs initially harvest plasma Fn and assemble it into early ECM already within the first 24 hours.

(a) Merged images of hMSC-assembled Fn ECM (fluorescence image) with brightfield images of hMSCs cultured for 24 hours on glass coverslips in growth medium supplemented with Fn-FRET at low (1×10^3 cells/cm^2) seeding densities.

(b) FRET false colors of hMSC-assembled Fn ECM at low (1×10^3 cells/cm^2) seeding densities. The FRET false color scheme represents the relative stretching of Fn fibrils with a color range of red to blue indicating folded to completely unfolded states of Fn, respectively.

(c) Histograms of Fn-FRET I_A/I_D ratios of hMSC-assembled Fn ECM in 24 hour cultures.

Scale bars: 50μm.

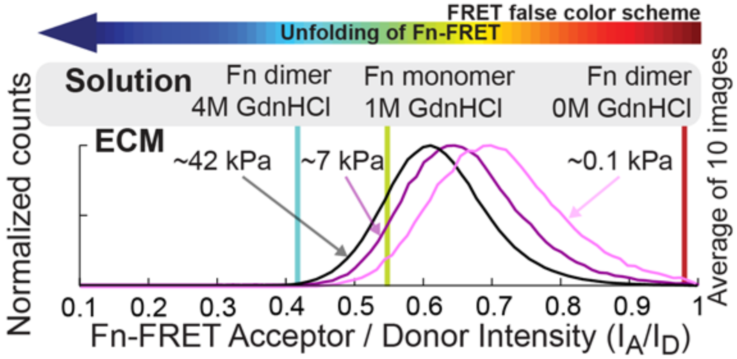

Figure S2: Stretching of Fn matrix increases with higher substrate rigidity. Related to Figure 2.

hMSCs were cultured in mixed induction medium supplemented with Fn-FRET and excess unlabeled Fn at an initial seeding density of 3×10^3/cm^2 on Fn-functionalized polyacrylamide substrates of high (~42kPa, black curve), medium (~7kPa, purple curve) and low (~0.1kPa, pink curve) rigidity. Confocal microscopic images of donor and acceptor peak intensities and differential interference contrast images (DIC) were taken 2μm above the glass-cell interface, followed by image analysis using MATLAB. Histograms of average I_A/I_D ratios of Fn matrices (average of 10 images of each rigidity) are shown. Solution denaturation values for dimeric Fn-FRET in 0M GdnHCl, monomeric Fn-FRET in 1M and dimeric Fn-FRET 4M GdnHCl are shown as red, green and blue lines respectively.

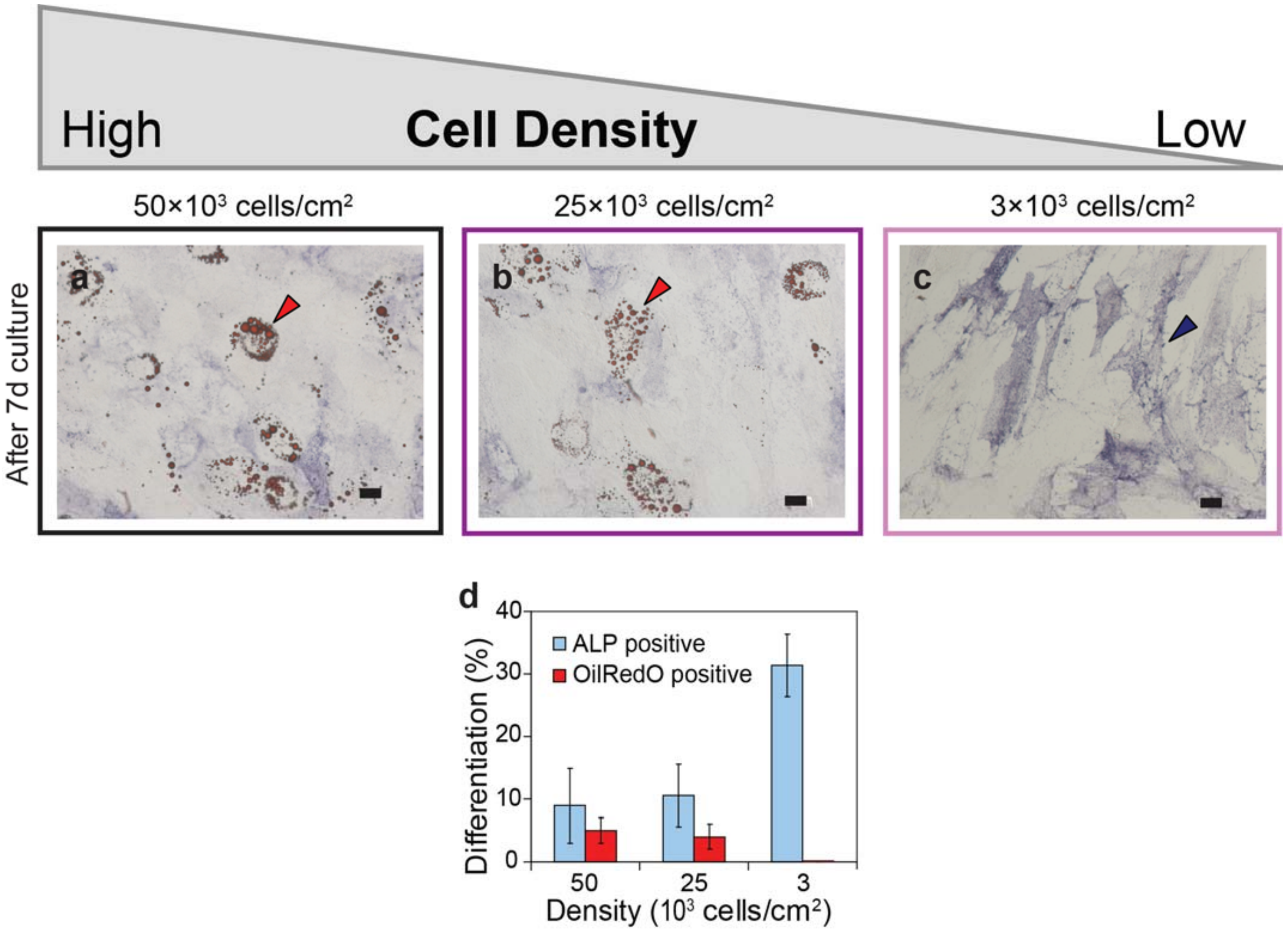

Figure S3: Higher cell density decreases osteogenesis and increases adipogenesis of hMSCs in 2D.

(a-c) Brightfield images of hMSCs cultured at seeding densities of 50×10^3 (a), 25×10^3 (b) or 3×10^3 cells/cm^2 (c) for 7 days on glass coverslips in mixed induction medium supplemented with Fn-FRET (5 µg/ml Fn-FRET, 45 µg/ml unlabeled Fn), with histochemical staining for ALP (blue, blue arrows) and OilRedO (red, red arrows). Scale bars: 50µm.

(d) Percentage of OilRedO and ALP positive hMSCs. OilRedO positive cells were mostly suppressed at a seeding density of 3×10^3 cells/cm^2. Results are shown as the mean ± s.d. (n=3).

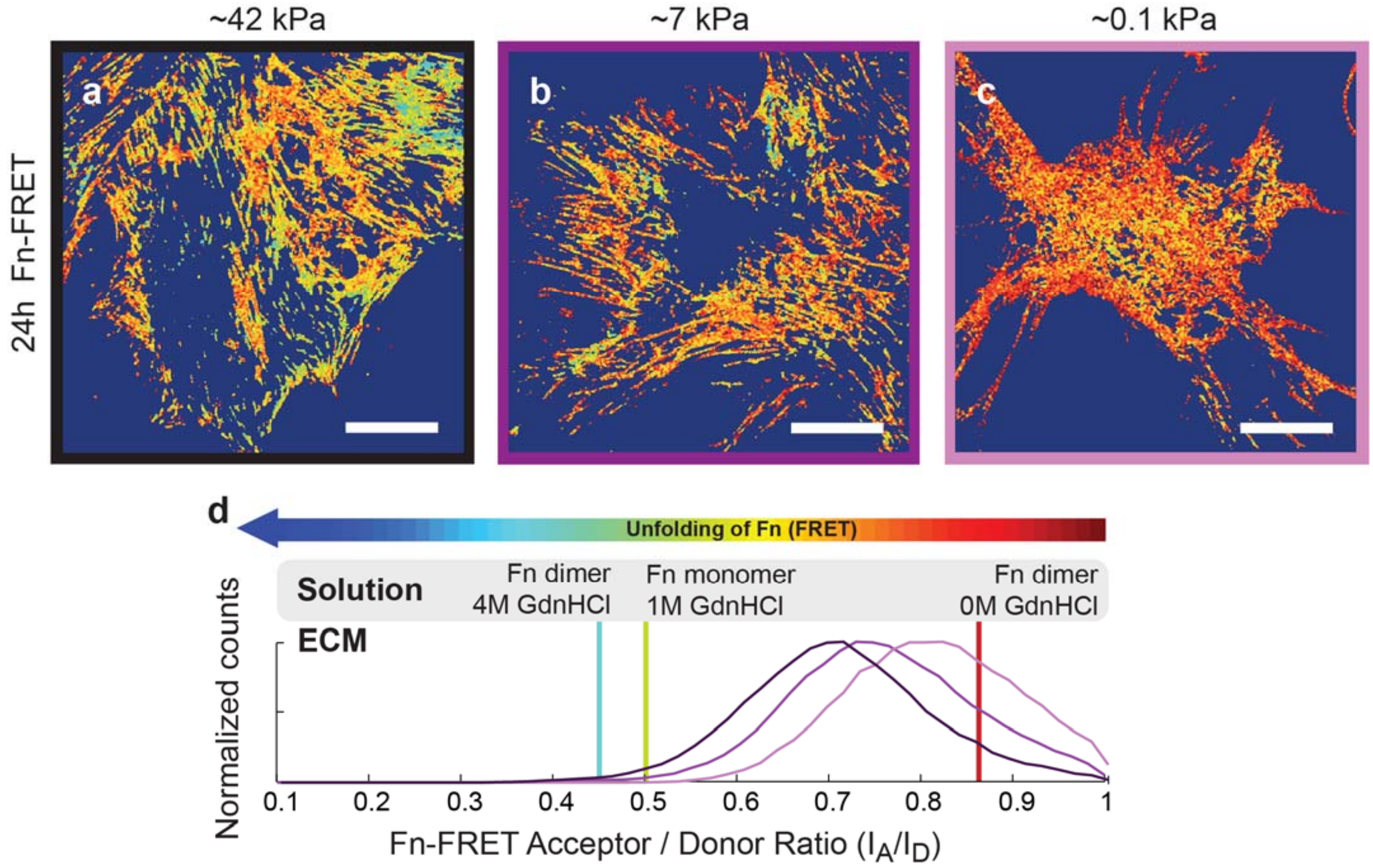

Figure S4: Stretching of early Fn matrix fibrils correlates with the rigidity of collagen I-functionalized polyacrylamide gels.

hMSCs were cultured in mixed induction medium supplemented with Fn-FRET and excess unlabeled Fn at an initial seeding density of $3\times10^3/cm^2$ on collagen I-functionalized polyacrylamide substrates of high (a, black curve), medium (b, purple curve) and low (c, pink curve) rigidity. Histograms of I_A/I_D ratios of Fn matrices are shown in d. Solution denaturation values for dimeric Fn-FRET in 0M GdnHCl, monomeric Fn-FRET in 1M and dimeric Fn-FRET 4M GdnHCl are shown as red, green and blue lines respectively.

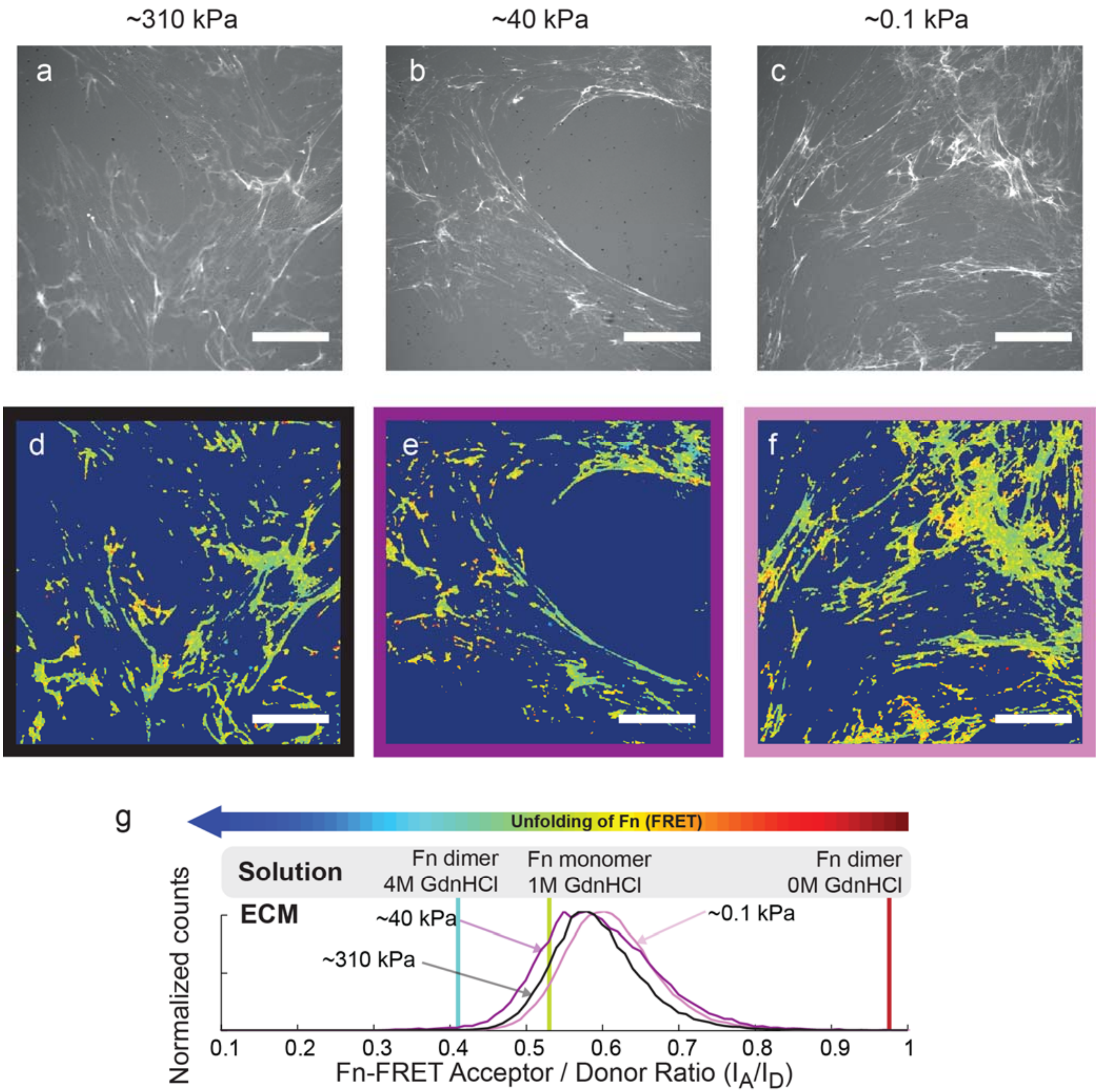

Figure S5: hMSCs cultured on PDMS substrates of varying rigidity which are covalently crosslinked to Fn as previously described [1].

(a-c) Merged images of hMSC-assembled Fn matrices (fluorescence image) with brightfield images of hMSCs cultured on rigid (a, ~310 kPa), medium (b, ~40 kPa) or soft (c, ~0.1 kPa) Fn-functionalized PDMS substrates at a seeding density of 3×10^3 cells/cm^2 for 24 hours in growth medium supplemented with trace amounts of Fn-FRET.

(d-f) FRET false colors of hMSC assembled Fn matrices on rigid (d), medium (e) or soft (f) Fn-functionalized PDMS substrates. Scale bars: 50µm.

(g) Histograms of Fn-FRET I_A/I_D ratios of hMSC assembled 24 hour Fn matrices on rigid (black curve), medium (purple curve) or soft (pink curve) Fn-functionalized PDMS substrates. Solution denaturation values for dimeric Fn-FRET in 0M GdnHCl, monomeric Fn-FRET in 1M and dimeric Fn-FRET 4M GdnHCl are shown as red, green and blue lines respectively.

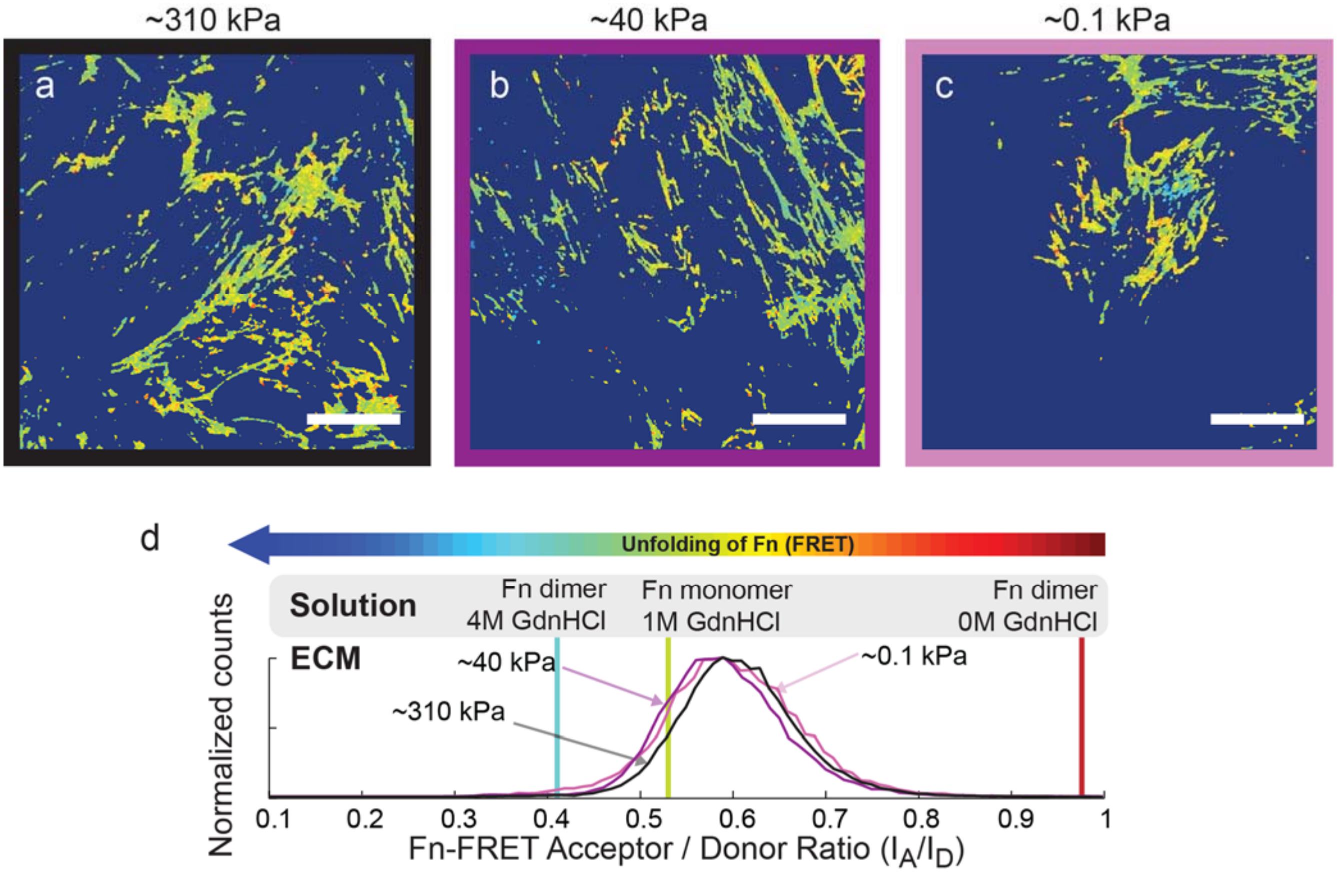

Figure S6: hMSCs cultured on PDMS substrates of varying rigidity which are crosslinked to collagen I. (a-c) Fn-FRET false colors of hMSC assembled Fn matrices on rigid (a), medium (b) or soft (c) Collagen I-functionalized PDMS substrates. Scale bars: 50µm.

(d) Histograms of Fn-FRET I_A/I_D ratios of hMSC assembled 24 hour Fn matrices on rigid (black curve), medium (purple curve) or soft (pink curve) Collagen I-functionalized PDMS substrates. Solution denaturation values for dimeric Fn-FRET in 0M GdnHCl, monomeric Fn-FRET in 1M and dimeric Fn-FRET 4M GdnHCl are shown as red, green and blue lines respectively.

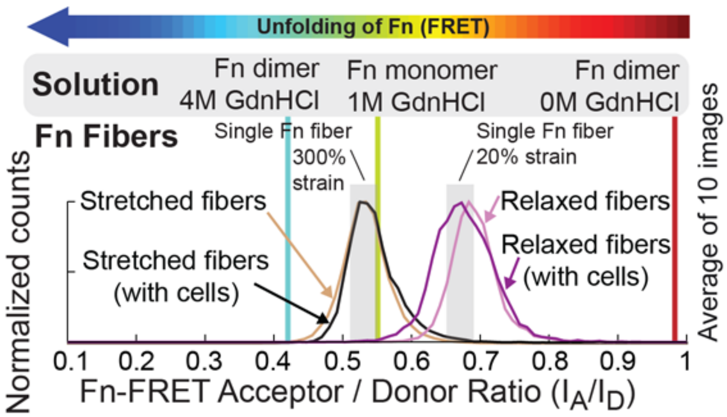

Figure S7: Histograms of the average Fn-FRET I_A/I_D ratios of 10 relaxed fibers before (pink curve) and after cell were cultured for 2 days on these fibers (purple curve), and 10 stretched fibers before (brown curve) and after (black curve). Fiber strains corresponding to 20% and 300% strain are shown as gray bars. Solution denaturation values for dimeric Fn-FRET in 0M, monomeric Fn-FRET in 1M and dimeric Fn-RET in 4M GdnHCl are shown as red, green and blue vertical lines respectively.

Supplemental References

1. Trappmann, B. *et al.* Extracellular-matrix tethering regulates stem-cell fate. *Nat. Mater.* **11**, 642-649 (2012).
